# Sepsis-Associated Encephalopathy: From Delirium to Dementia?

**DOI:** 10.3390/jcm9030703

**Published:** 2020-03-05

**Authors:** Ha-Yeun Chung, Jonathan Wickel, Frank M. Brunkhorst, Christian Geis

**Affiliations:** 1 Section Translational Neuroimmunology, Department of Neurology, Jena University Hospital, 07747 Jena, Germany; ha-yeun.chung@med.uni-jena.de (H.-Y.C.); jonathan.wickel@med.uni-jena.de (J.W.); 2 Center for Sepsis Control and Care, Jena University Hospital, Jena 07747, Germany; frank.brunkhorst@med.uni-jena.de; 3 Center for Clinical Studies and Department of Anesthesiology and Intensive Care Medicine, Jena University Hospital, 07747 Jena, Germany

**Keywords:** sepsis, encephalopathy, brain dysfunction, SAE, delirium, dementia, cognitive deficits, pathophysiology, long-term sequelae

## Abstract

Sepsis is a major cause of death in intensive care units worldwide. The acute phase of sepsis is often accompanied by sepsis-associated encephalopathy, which is highly associated with increased mortality. Moreover, in the chronic phase, more than 50% of surviving patients suffer from severe and long-term cognitive deficits compromising their daily quality of life and placing an immense burden on primary caregivers. Due to a growing number of sepsis survivors, these long-lasting deficits are increasingly relevant. Despite the high incidence and clinical relevance, the pathomechanisms of acute and chronic stages in sepsis-associated encephalopathy are only incompletely understood, and no specific therapeutic options are yet available. Here, we review the emergence of sepsis-associated encephalopathy from initial clinical presentation to long-term cognitive impairment in sepsis survivors and summarize pathomechanisms potentially contributing to the development of sepsis-associated encephalopathy.

## 1. Introduction

Sepsis is a life-threatening and multi-factorial disease with continuously increasing incidence over recent decades from 300 to 1000 sepsis cases per 100,000 people per year in the United States. The overall global incidence is approximately 31 million sepsis cases per year [1,2].

Sepsis-associated encephalopathy (SAE) is one of the most common complications during the acute phase and in later stages after surviving sepsis. It is defined by a diffuse cerebral dysfunction due to the dysregulated host response and absence of a direct central nervous system (CNS) infection. SAE symptoms may already be present before sepsis criteria are fulfilled. Symptoms in the acute stage range from sickness behavior and delirium to coma and may later result in long-term cognitive impairment [3,4]. As SAE is a diagnosis of exclusion, other causes of encephalopathy (e.g., metabolic changes, drug intoxication, structural brain lesions, cerebrovascular events, encephalitis, meningitis, and non-convulsive status epilepticus) need to be ruled out in sepsis patients [5]. From a clinical point of view, the disease course of SAE can be sub-divided into an acute and a chronic phase.

### 1.1. Acute Phase of SAE—Delirium

The acute phase of SAE is mainly characterized by symptoms of delirium with (sub-)acute changes of the patient’s consciousness. Among others, symptoms include agitation, hallucinations, reduced concentration, and alteration of the sleep–wake cycle [3]. Depending on the disease severity, patients may become somnolent or even comatose. Similar neurological and psychiatric manifestations can also be found in encephalopathies of other etiologies, e.g., encephalopathy due to organ failure, intoxication, or vitamin deficiency. Nonetheless, in encephalopathy due to organ failure or vitamin deficiency, additional symptoms may occur, e.g., asterixis or ataxia and eye movement disorders. Patients with features of encephalopathy presenting such additional symptoms should undergo clinical re-evaluation, since these symptoms may point towards a metabolic etiology, e.g., hepatic encephalopathy or uremia or hypovitaminosis [6,7].

In a recent retrospective analysis of a large multi-center database, the incidence of SAE was identified to be higher in sepsis patients with pre-existent cognitive deficits, long-term use of psychoactive drugs, neurological diseases, and metabolic alterations during sepsis (e.g., hypoglycemia, hyperglycemia, hypercapnia, hypernatremia) [8]. However, this study had several limitations, including the definition of SAE, which was based on the patient’s Glasgow coma scale (GCS) at admission to the ICU. Therefore, a definitive statement for a causal relationship between development of SAE and possible risk factors could not be provided [8].

Earlier studies have already suggested a high association of SAE and mortality of sepsis patients by emphasizing mortality rates over 60% in these patients [9]. Even mild alterations of the mental status (GCS of 13–14) seem to have prognostic potential towards a worse outcome in sepsis [8]. Delirium due to SAE correlates with the development of long-term cognitive dysfunction following hospital discharge [10,11,12,13]. Of note, the length of delirium is the only proven risk factor for the development of long-term cognitive dysfunction so far and may also be associated with lower brain volume [10,12,14,15]. Against this, mechanical ventilation, hypoxia, sedatives, intra-operative hypotension, and use of analgesics were not associated with long-term cognitive dysfunction [16,17,18,19,20]. However, most of the previously mentioned studies did not distinguish between sepsis survivors and survivors following critical illness in general. Therefore, no final statement of the exact risk factors contributing to poor neurocognitive outcome of sepsis patients can be given.

### 1.2. Chronic Phase of SAE—Dementia

Due to an increasing incidence of sepsis and a decrease in mortality rates, the number of sepsis survivors is steadily growing. Nevertheless, survival of sepsis is often accompanied by long-term cognitive impairment [21,22,23,24]. More than half of sepsis survivors suffer from cognitive dysfunction predominantly affecting general memory, attention, verbal fluency, and executive function at hospital discharge [14]. In a significant proportion of patients, cognitive dysfunction can even reach the degree of mild Alzheimer’s disease (mild cognitive impairment, MCI) [15]. In addition to cognitive dysfunction, the incidence of psychiatric disorders, e.g., depression, anxiety, post-traumatic stress disorder, and tendency to self-harm is higher in sepsis survivors than in the general population [25,26]. Thus, similar to patients surviving critical illness, sepsis survivors have a significantly reduced quality of life [27]. Even though long-term consequences after sepsis are well known, no treatment or standardized management suggestions are proposed in the current medical guidelines so far.

Furthermore, neurocognitive dysfunction and long-term sequelae after critical illness cause a major burden to the health care system with high socio-economic costs [28]. These costs emerge from an increased risk of repeated hospitalization, accommodation in nursing homes, reduced working capacities of informal caregivers, and premature death [22,29,30].

## 2. Epidemiology

SAE is the most common cause for encephalopathies in ICUs worldwide [3], affecting up to 50% of patients during the course of sepsis [8,9]. A proven bacteremia even increases the incidence of SAE up to 70%. Patients with renal, liver, or multi-organ failure are more frequently affected than sepsis patients without organ complications [9]. The mortality rate of sepsis patients increases from 26% to 49% with SAE and is associated with higher values of the GCS, sequential organ failure assessment score (SOFA), and the APACHE II score [8,31,32]. At hospital discharge, almost 45% of sepsis survivors show symptoms of long-term cognitive dysfunction [15]. However, an exact evaluation of incidence, prevalence, and mortality is restricted due to lack of definite SAE diagnosis criteria in these studies and due to the variable clinical presentation of SAE. A prospective cohort study with a clear definition of SAE is needed to address current uncertainties in incidence, severity, and outcome of SAE in acutely ill sepsis patients.

## 3. Pathophysiology

**Figure 1:**
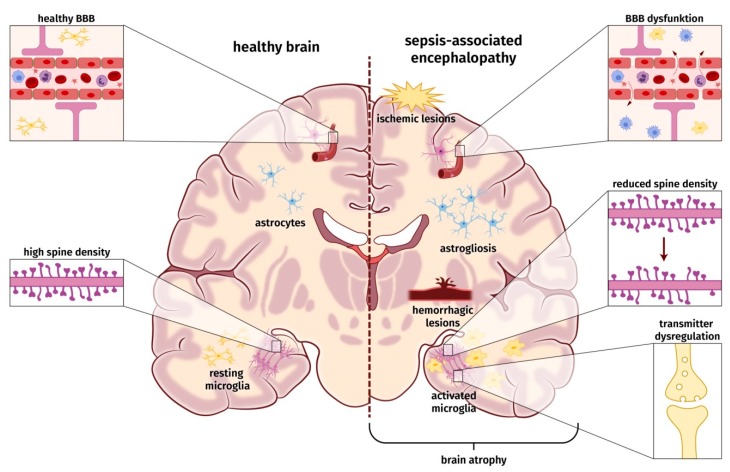
Schematic overview of probable pathophysiological and molecular alterations underlying sepsis-associated encephalopathy.

According to SAE definition, an acute infection of the CNS must be ruled out to diagnose SAE. Thus, SAE is regarded as a consequence of the dysregulated host response induced by severe systemic infection. So far, the pathophysiology and underlying molecular mechanisms leading to SAE are only incompletely understood. The etiology of SAE is likely multi-factorial, and a number of pathomechanisms are involved in parallel, influence each other, and contribute to a varying degree to the development of SAE [3]. These factors may involve ischemic/hemorrhagic lesions, a compromised blood–brain barrier (BBB), neuroinflammatory processes, e.g., microglia activation and astrogliosis, changes in neuronal synaptic spine density, and dysregulation of neurotransmitter (Figure 1). None of these factors seems to be obligatory to cause SAE.

### 3.1. Ischemic and Hemorrhagic Cerebral Lesions

Sepsis impairs both the macro-circulation and the micro-circulation of the brain. MRI studies, post-mortem analysis of sepsis patients, and also animal experiments confirm macro- and microscopic areas with ischemic and hemorrhagic lesions [33,34]. Hypotensive episodes during sepsis and septic shock result in decreased cerebral perfusion, which has been shown in several clinical studies [35,36]. In addition, disturbed systemic vasoreactivity and dysregulated autoregulation of cerebral arteries contribute to reduced cerebral perfusion [35,37]. Physiologically, cerebral autoregulation controls constant brain perfusion by regulating vasoconstriction of cerebral arteries. Endothelial dysfunction during sepsis leads to inconsistent cerebral blood flow, especially during blood pressure fluctuations. A disturbed autoregulation was present in almost 50% of sepsis patients with SAE [38]. However, this study was limited to evaluation of blood perfusion in large intra-cranial arteries, whereas dysfunction in the microcirculation was not analyzed [38]. Recent animal experiments in sheep revealed impaired cerebral microcirculation during septic shock resulting in decreased cerebral oxygenation [39]. In addition, coagulation disturbances might contribute to thrombotic occlusion of capillaries, resulting in neuronal anoxia and apoptosis [40].

### 3.2. Impairment of Blood–Brain Barrier and Neuroinflammation

Under physiological conditions, the BBB is essential for the maintenance of a constant extracellular milieu enabling normal neuronal function [41,42]. The BBB comprises endothelial cells, astrocytes, and pericytes composing a highly efficient border between the brain parenchyma and cerebral circulation [41]. During sepsis-induced dysregulated host response, proinflammatory cytokines, such as TNF-α and IL-1β, activate endothelial cells. Endothelial activation results in production of reactive oxygen species (ROS) and consequently in increased endothelial permeability [40,43]. In addition, activated endothelial cells induce the expression of adherence proteins. These proteins support the transmigration of activated immune cells through the impaired BBB into the CNS [41,44]. The increased release of ROS also harms macromolecules of the BBB, leads to mitochondrial dysfunction and activates matrix–metalloproteases. Subsequently, the BBB is impaired, and resident cells in the brain, e.g., astrocytes and microglia (neuroglia), can be activated. Neuroglia are responsible for the maintenance of cerebral homeostasis [45]. Under physiological conditions, astrocytes mediate a wide range of important regulatory functions in the CNS, including ionostasis, neurotransmitter metabolism, fluid balance, neurogenesis, and maintenance of synaptic plasticity [46]. The latter might be controlled by astrocyte-secreted synapse-modifying factors, such as hevin, SPARC, and TNF-α [47,48,49]. Experimental studies consistently describe a reactive astrogliosis as a result of the dysregulated immune response during the course of SAE [4,45]. The release of pro-inflammatory mediators and ROS by these cells may further aggravate the impairment of the BBB. At distinct areas, which are called the circumventricular organs, the BBB is physiologically leakier, and crossing of cytokines to into the CNS is facilitated.

Microglia, as the brain’s resident mononuclear cells of the innate immune system, play a key role in protecting the brain from neuronal damage. In their resting state, microglia are essential for physiological, non-inflammatory surveillance functions and regulation of synaptic spines during development and are also critically involved in the regulation of synaptic plasticity [50,51]. Microglia are activated in the course of sepsis and might contribute to aberrant neuronal function and loss of dendritic spines in CA1 hippocampal pyramidal neurons [52,53,54]. There is growing evidence that activation of microglia may result from infiltrating peripheral monocytes bypassing the dysregulated BBB and induce long-term activation of resident microglia after sepsis [55,56].

### 3.3. Dysregulated Neurotransmitter

Several neurotransmitters are discussed to be involved in the development and maintenance of SAE. Most studies so far have highlighted dysregulations of cholinergic pathways, but experimental studies also suggest that gamma-aminobutyric acid, norepinephrine, serotonin, and dopamine pathways are compromised [57,58]. Massive cytokine release in sepsis might cause a dysregulation of neurotransmission in experimental sepsis [59]. Due to these experimental findings and previous clinical observations that anti-cholinergic medication worsens delirium [60], it has been postulated that an affected cholinergic pathway might induce delirium in critically ill patients [57]. However, a double-blind and placebo-controlled study revealed that treating ICU patients with rivastigmine—a cholinesterase inhibitor—results in even longer delirium and increased mortality, leading to an early study termination [61]. Even though this study was not designed to investigate the effect of rivastigmine on SAE patients, these results suggest that cholinesterase inhibitors do not have a beneficial effect in sepsis patients. A previous study found a decreased concentration of tyrosine, tryptophan, and phenylalanine (amino acids essential for the neurotransmitter synthesis) in the serum of sepsis patients [62]. However, it is unclear how these observational and partly controversial data are causative related to the development of SAE.

## 4. Neuropathological Findings

Important information of SAE pathophysiology can be obtained from autopsy studies. Autopsy in twelve patients with encephalopathy and bacterial infection revealed disseminated brain micro-abscesses (in eight patients) and proliferation of astrocytes and microglia in the cerebral cortex (in four patients) [63]. Nonetheless, it should be noted that existence of cerebral (micro-)abscesses should rather be regarded as infectious encephalitis than as SAE. Additionally, cerebral infarcts, brain purpura, multiple small white matter hemorrhages, and central pontine myelinolysis were described [63]. Another post-mortem brain analysis of 23 patients who died due to septic shock revealed hemorrhages (26%), signs of hypercoagulability (9%), micro-abscesses (9%), multi-focal necrotizing leukoencephalopathy (9%) and ischemic lesions (100%). Ischemia affected predominantly autonomic centers [33]. A prospective cohort study with post-mortem examinations found an increase of neuronal and glial apoptosis in autonomic centers, e.g., supra-optic and paraventricular nuclei, cerebral amygdala, locus coeruleus, and medullary autonomic nuclei. The lesions were likely triggered by inducible nitric oxide synthase [64]. In another post-mortem analysis of three patients who died from septic shock, marked lesions of the pons and typical lesions of multi-focal necrotizing leukoencephalopathy were described [65].

In respect of microglia, a case control study found an increase of microglia in the grey matter [53]. A following prospective post-mortem study showed an increase of CD-68 positive microglia in the putamen, hippocampus, and cerebellum compared to patients who died due to other diseases [66]. In contrast, in another post-mortem study, the microglia activation was more pronounced in the white matter compared to the grey matter [67].

## 5. Diagnostic Procedures

**Figure 2:**
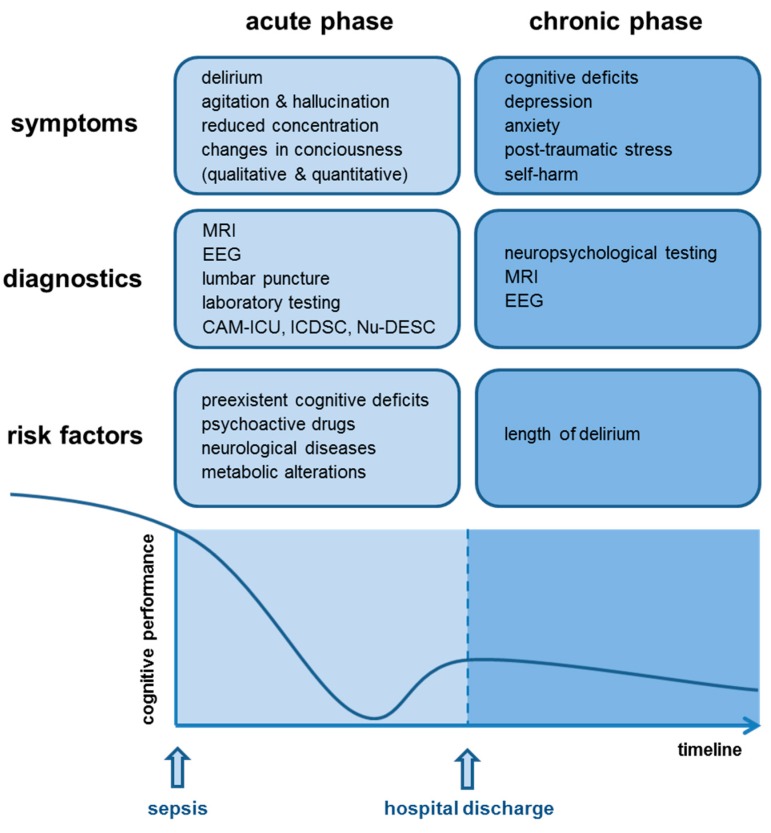
Symptoms, diagnostics, risk factors, and schematic overview of the cognitive performance in acute and chronic sepsis-associated encephalopathy (SAE).

Since the diagnosis of SAE is a diagnosis of exclusion, a broad range of diagnostic tests must be performed to ensure that primary cerebral pathologies are excluded [5] (Figure 2). Since the acute phase of SAE is developing during acute illness and infection, SAE diagnosis is often delayed due to sepsis complications (hypoxemia, electrolyte disorder, liver and renal failure) or the use of sedatives.

### 5.1. Cerebral Imaging

Conventional computed tomography (CT) and magnetic resonance imaging (MRI) are most frequently used for brain imaging. In critically ill patients, a cerebral CT scan is primarily used to exclude intra-cranial brain edema and ischemic or hemorrhagic lesions. Nonetheless, except for hemorrhagic lesions, MRI has a higher sensitivity for the detection of structural lesions and is therefore preferred for cerebral imaging [68].

Despite severe symptoms, in 52% of SAE patients, cerebral imaging is unremarkable, especially in acute stages. Pathological imaging findings are usually unspecific and also occur in other diseases unrelated to sepsis [4]. In a prospective observational study, MRI scans in acute stages of SAE identified ischemic lesions (29%) as the most common pathological findings [34]. These lesions are displayed in diffusion-weighted imaging and represent cytotoxic edema usually caused by ischemia, hypoxia, or vasogenic edema and may indicate circulatory impairment (e.g., impaired macro- and micro-circulation) [68]. In addition, ischemic stroke was independently associated with increased mortality and poor neurologic outcome [34]. In approximately 21% of sepsis patients, leukoencephalopathy can be found in MRI, which might be a possible marker of BBB leakage [34]. In this study, however, only a highly selective patient population fulfilling the criteria of septic shock and demonstrating severe neurological symptoms (coma, delirium, focal neurologic deficit or seizure) was included. There is no information about the prevalence of MRI lesions in less severely affected or neurologically asymptomatic sepsis patients. In some cases, signs of a posterior reversible encephalopathy syndrome can be detected [69]. In a recent prospective MRI neuroimaging study, sepsis survivors revealed a global and/or partial atrophy with mesial temporal emphasis up to 12 months following hospital discharge. Due to the limited sample size, a general statement regarding the frequency and the degree of SAE induced atrophy is limited [12].

### 5.2. Electroencephalography

Several case series and small studies describe a variable prevalence of electroencephalography EEG) abnormalities (ranging from 12% to 100%), e.g., appearance of theta and delta waves [34,60,70]. This high range of abnormal EEG findings might be due to the small sample size and heterogeneity of the study population. Severe EEG abnormalities with periodic and rhythmic discharges (e.g., triphasic waves, frontal intermittent rhythmic delta activity, general periodic discharges) may indicate severe SAE. Sometimes, epileptiform discharges (e.g., periodic lateralized, bilateral independent lateralized) can be recorded [34,60,70,71]. In sepsis patients with bacteremia but no detectable cognitive deficits, abnormalities in EEG could be demonstrated in even 50% of cases [70]. Thus, EEG as a non-invasive investigation tool is helpful to assess the severity of SAE, as it reflects the degree of encephalopathy in general. Absence of EEG modulation, a delta-predominant background, and periodic discharges might be independent predictors of mortality and were also associated with the occurrence of delirium in sepsis patients [72,73]. In addition, seizures occur in almost 10%–20% of sepsis patients, most commonly non-convulsive seizures [71,72]. At this point, seizures should be treated with anti-convulsants, as they worsen the outcome in critically-ill patients [74]. However, it is important to note that none of these EEG abnormalities are specific for SAE and can also widely occur in encephalopathy of other etiology.

### 5.3. Laboratory Testing—Cerebrospinal Fluid

Surprisingly few studies exist to evaluate specific cerebrospinal fluid (CSF) biomarkers related to the diagnosis and prediction of long-term outcome of SAE. Clinical routine laboratory testing of CSF is restricted to the exclusion of other etiologies of encephalopathy, such as meningitis and encephalitis. Findings in SAE are limited to a slight protein increase as a sign of local inflammation or impairment of BBB without specific intrathecal immunoglobulin synthesis.

### 5.4. Laboratory Testing—Blood

Due to numerous complications in patients with SAE, routine laboratory tests are essential, including complete blood cell count, electrolytes, and organ function parameters to identify possible modifiable risk factors [8]. There exists no validated biomarker for prediction or confirmation of SAE so far. In several studies, a serum increase of neuron-specific enolase (NSE) in 53% and S100b in 42% in sepsis patients could be detected [75]. S100b is a marker of glial cell damage, whereas NSE is a marker for neuron damage. Serum concentration of these markers was also associated with brain injury and neurological impairment [75,76]. However, screening for S100b and NSE for the diagnosis of SAE or use as a prognostic marker is not recommended due to inconsistent study results. Very recent studies indicate a higher specificity and sensitivity for increased detection of neurofilaments, especially the light chain of neurofilaments (NFL light chain), in the course of SAE [77]. Neurofilaments are essential structural proteins in neurons mainly located in the axonal cytoplasm. In response to neuronal damage, the concentration of NFL light chain increases in serum as well as CSF and can be measured using single-molecule array technology (SiMoA) [78]. Serum levels of NFL light chain are currently evaluated for diagnostic, prognostic, and monitoring purposes in a variety of neurological diseases [79,80]. These promising results of NFL light chain serum concentrations in sepsis and their predictive value for SAE need to be evaluated prospectively. It will be interesting to see if these changes correlate to the development of cognitive dysfunction in the late phase of SAE.

### 5.5. Screening Tests for Delirium in Acute SAE

Early detection of delirium is of great importance, since delirium can be the first symptom of sepsis and can even precede the fulfillment of sepsis criteria [3]. In the management of SAE, a fast and sufficient treatment of the underlying infection, the control of organ dysfunction, and metabolic alterations is essential.

There are several screening tests available to test for delirium depending on the patient’s condition. In the intensive care unit, the CAM-ICU [81] or the intensive care delirium checklist (ICDSC) [82] are most commonly used. The specificity of the CAM-ICU is 0.97, and the sensitivity is 0.79 [83]. As the ICDSC has a higher sensitivity (0.99), it may be used as a screening tool and, if delirium is suspected, the CAM-ICU may be additionally performed to confirm delirium [83].

In less severely affected patients, the confusion assessment method (CAM) [84] is regarded as the gold standard. There are two modified versions of the CAM: The 3D-CAM (sensitivity 0.95; specificity 0.94) [85], which can be performed within three minutes, and the CAM-S, which additionally evaluates the severity of delirium [86]. Alternatively, the nursing delirium screening scale (Nu-DESC) [87] or the 4 AT test [88] can be used to detect delirium.

Despite the availability of several screening tests, clinical routine screening for delirium is insufficiently applied on a regular basis. It has been shown that only 27% of ICU patients were regularly screened with a validated delirium screening tool [89]. This proportion is even lower in less severely affected patients on the general ward. As hypoactive delirium is likely to be missed without regular screening, there is need for increased awareness and more intense monitoring.

## 6. Therapeutic Management

### 6.1. Pharmacological Treatment

Although numerous pharmacological treatment strategies have been studied in recent decades, no evidence-based pharmacological treatment option is available which is able to convincingly demonstrate effects on delirium in SAE [90]. Therefore, in clinical routine, no specific recommendation for a standardized pharmacological treatment can be given. It is unclear so far whether delirium as a manifestation of acute SAE should be treated with additive use of pharmacological substances, e.g., neuroleptic drugs [91]. In a randomized, double-blind, placebo-controlled trial, commonly used neuroleptic drugs, such as haloperidol and ziprasidone, were not able to shorten delirium-free days as compared to placebo in ICU patients [92]. This was confirmed by a recent systematic review showing that the use of highly potent neuroleptic agents, e.g., haloperidol or second-generation anti-psychotics, was not favorable regarding mortality, delirium severity, hospital length of stay, or cognitive function in delirium [93].

Since in the management of SAE a fast and sufficient treatment of the underlying infection, control of organ dysfunction, and metabolic alterations (e.g., hypoglycemia, hyperglycemia, hypercapnia, hypernatremia) is essential, delirium as a first presentation of sepsis must not be overlooked. Additionally, numerous commonly used drugs in the ICU reveal neurotoxic side effects that may trigger or maintain delirium. These include drugs with anti-cholinergic, histaminergic or psychotropic effects [94]. Pharmacological vigilance and non-pharmacological strategies should support delirium treatment, which has already been evaluated in small cohort studies. If possible, benzodiazepines and opioids should be avoided, as they are independent risk factors for the development of acute SAE at the ICU [8]. Furthermore, in a sub-group analysis, the alpha-2 agonist dexmedetomidine revealed significant advantages regarding delirium-free days, shortening of mechanical ventilation, and decreased mortality rates in sepsis patients as compared to lorazepam [95]. However, these results could not be replicated in a multi-center randomized clinical [96]. A current randomized controlled trial evaluates the benefit of an early use of alpha-2 agonists (dexmedetomidine and clonidine, respectively) in mechanically-ventilated patients compared to use of propofol (NCT03653832). Additionally, there is evidence that starting a statin therapy in sepsis patients might lower daily risk of delirium, whereas stopping a pre-existing statin therapy might increase delirium risk [97,98].

### 6.2. Non-Pharmacological Treatment

As there are so far no sufficient pharmacological treatment options, non-pharmacological treatment strategies are important and should be implemented in ICU patients and in less severely affected sepsis patients. These comprise a strict sleep protocol, occupational therapy with cognitive stimulation, use of glasses and hearing aid, early mobilization, as well as devices for orientation, such as clock, television, radio, pictures, and music therapy [99,100,101].

## 7. Conclusions

The development of SAE is an acute and frequent complication of the dysregulated host response during the acute phase of sepsis, which often results in long-term cognitive deficits in sepsis survivors. This underlines the importance for an early screening for delirium or other SAE symptoms by trained medical staff. This is particularly important as SAE may precede clinical signs of sepsis, and early treatment may improve the neurocognitive outcome. In addition to source control of the infectious focus and antibiotic treatment, modifiable risk factors for delirium should be identified. So far, specific pharmacological delirium treatment is not available, and there is an urgent need to develop, to evaluate, and to finally implement effective therapeutic options to treat SAE.

Future experimental and clinical studies should focus on both the acute and chronic stages of SAE. We have to proceed with experimental and clinical research to elucidate the complex pathophysiological and molecular mechanisms in acute and chronic SAE to be capable of developing novel and specific concepts for preventing and treating SAE. Evaluating the role of neuroglia activation and BBB disruption in SAE may be especially promising, since this offers the possibility of a targeted intervention in the development of SAE in the acute stage. In clinical terms, it would be worthwhile to establish valid biomarkers, e.g., use of serum levels of NFL light chain in the acute stage or innovative imaging procedures for prognostic estimation of later neurocognitive dysfunction. In the post-acute stages, effects of individualized cognitive training need to be tested. Therefore, there is an urgent need for prospective and controlled clinical trials in SAE patients to expand empirical knowledge towards evidence-based medical interventions.
